# Electrical and photo-electrical properties of MoS_2_ nanosheets with and without an Al_2_O_3_ capping layer under various environmental conditions

**DOI:** 10.1080/14686996.2016.1167571

**Published:** 2016-04-08

**Authors:** Muhammad Farooq Khan, Ghazanfar Nazir, Volodymyr M. lermolenko, Jonghwa Eom

**Affiliations:** ^a^ Department of Physics & Astronomy and Graphene Research Institute, Sejong University, Seoul05006, Korea

**Keywords:** MoS_2_, transition metal dichalcogenides, electrical property, photoresponse parameters, Raman spectroscopy, DUV light, 40 Optical, magnetic and electronic device materials, 105 Low-Dimension (1D/2D) materials, 100 Materials, 201 Electronics / Semiconductor / TCOs, 200 Applications, 212 Surface and interfaces, 200 Applications

## Abstract

The electrical and photo-electrical properties of exfoliated MoS_2_ were investigated in the dark and in the presence of deep ultraviolet (DUV) light under various environmental conditions (vacuum, N_2_ gas, air, and O_2_ gas). We examined the effects of environmental gases on MoS_2_ flakes in the dark and after DUV illumination through Raman spectroscopy and found that DUV light induced red and blue shifts of peaks (E^1^_2 g_ and A_1 g_) position in the presence of N_2_ and O_2_ gases, respectively. In the dark, the threshold voltage in the transfer characteristics of few-layer (FL) MoS_2_ field-effect transistors (FETs) remained almost the same in vacuum and N_2_ gas but shifted toward positive gate voltages in air or O_2_ gas because of the adsorption of oxygen atoms/molecules on the MoS_2_ surface. We analyzed light detection parameters such as responsivity, detectivity, external quantum efficiency, linear dynamic range, and relaxation time to characterize the photoresponse behavior of FL-MoS_2_ FETs under various environmental conditions. All parameters were improved in their performances in N_2_ gas, but deteriorated in O_2_ gas environment. The photocurrent decayed with a large time constant in N_2_ gas, but decayed with a small time constant in O_2_ gas. We also investigated the characteristics of the devices after passivating by Al_2_O_3_ film on the MoS_2_ surface. The devices became almost hysteresis-free in the transfer characteristics and stable with improved mobility. Given its outstanding performance under DUV light, the passivated device may be potentially used for applications in MoS_2_-based integrated optoelectronic circuits, light sensing devices, and solar cells.

## Graphical abstract

**Figure:**
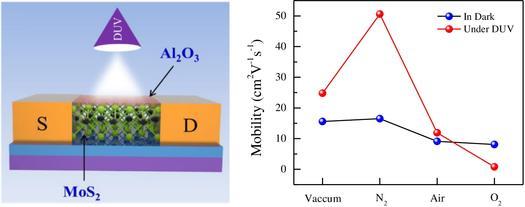

## 1. Introduction

Two-dimensional (2D) nanomaterials have sustained attention because of their unique properties and facile fabrication process despite the complexity of their structures. Graphene as a 2D material has become popular in recent years because of its outstanding linear dispersion relation, high charge carrier mobility, feasibility of chemical doping, and other unique physical properties conferred by its low dimensionality.[1–5] However, the zero band gap of graphene hinders its application in logic and optoelectronic devices. Graphene-based field–effect transistors (FETs) cannot be efficiently switched off; that is, off-current is comparable with on-current. However, 2D transition metal dichalcogenides (TMDCs) possess a suitable band gap of ~1–2 eV, making these materials potentially applicable in nanoelectronics, sensing, and photonics.[6–9] Several studies have analyzed 2D TMDC-based FETs, photodetectors, and gas sensors. The earliest TMDC, namely WSe_2_ crystal, was used in FETs and exhibits a high mobility (>500 cm^2^ V^−1^s^−1^) and ambipolar behavior with a ~10^4^ on/off ratio at 60 K.[10] TMDCs are potential materials for molecular sensing application because of their high surface-to-volume ratio. MoS_2_ sheets are sensitive detectors of NO, NO_2_, NH_3_, N_2_, and triethylamine gases.[11–15] However, the large variation in the electrical transport properties of MoS_2_ is generally attributed to extrinsic or environmental effects. These effects may significantly limit the exploration of the intrinsic properties of MoS_2_. Investigating the environmental factors that influence the reliability and stability of MoS_2_ FETs is important. Detailed and comparative investigations on environmental gases effects are needed to understand the overall performance of MoS_2_ FETs and undesirable effects should be minimized or removed to enhance device reliability and stability.

In this study, we fabricated few-layer (FL) MoS_2_ FETs and investigated electrical transport properties under various environmental conditions. We analyzed the change in on-state current under different environmental conditions. We observed the hysteresis behavior in transfer characteristics, where oxygen on the MoS_2_ surface plays a vital role in hysteresis. Gas environment significantly affects the characteristics of MoS_2_ FETs under deep ultraviolet (DUV) light. Thus, we investigated the time-dependent photoresponse of MoS_2_ FETs in the presence of various environmental gases. We also studied the maximum photocurrent saturation, responsivity, detectivity, external quantum efficiency (EQE), linear dynamic range (LDR), and relaxation time after switching light ON and OFF under various environmental conditions. Finally, we investigated the characteristics of devices after passivating by Al_2_O_3_ film on the MoS_2_ surface. The devices became almost hysteresis-free and stable FETs with improved mobility. The effect of Al_2_O_3_ on MoS_2_ flakes was discussed to explore the basic and important parameters regarding light detection.

## 2. Experimental section

**Figure 1.**
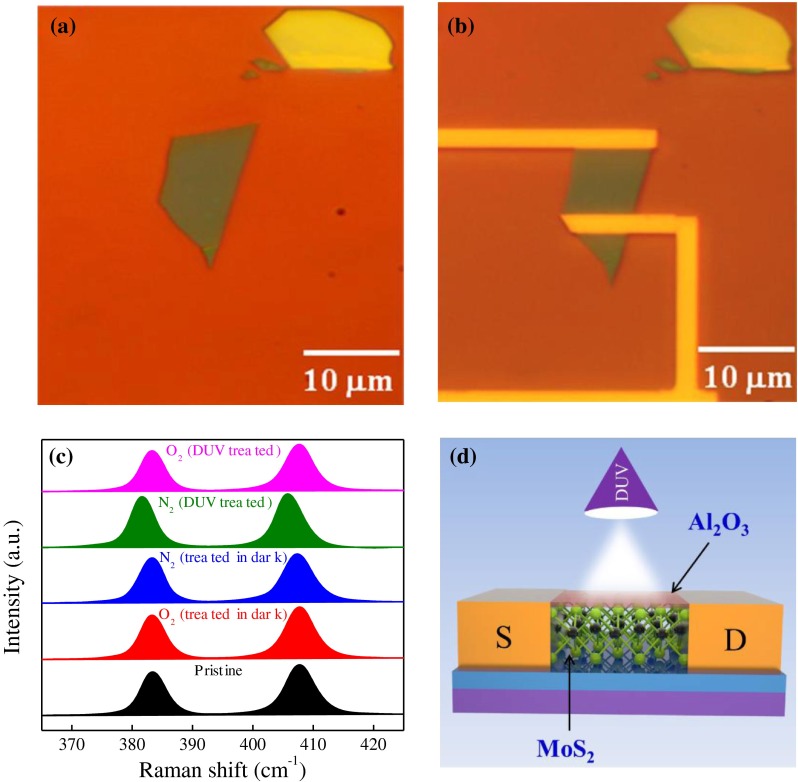
Few-layer MoS_2_ FET. (a) Optical image of FL-MoS_2_ flake serving as a conducting channel in the transistor. (b) Optical image of the final device based on the flake shown in (a). The two contacts were used for electrical transport measurements. (c) Raman spectra of the FL-MoS_2_ pristine (black), O_2_ treated in the dark (red), N_2_ treated in the dark (blue), N_2_ treated under DUV (green), and O_2_ treated under DUV (pink). (d) Schematic view of the structure of the passivated (10 nm Al2O3 layer) FL-MoS_2_ FET under DUV illumination. A FL-MoS_2_ was transferred on a p-doped silicon substrate with a 300-nm-thick SiO_2_ capping layer. The substrate served as a back gate.

We fabricated FL-MoS_2_ FETs using a conventional approach. FL-MoS_2_ flakes were transferred to clean 300-nm-thick SiO_2_ layer on p++ Si substrate with Scotch tape via mechanical cleavage method.[16] FL flakes were initially identified under an optical microscope (Figure 1(a)) and further confirmed through Raman spectroscopy. The thickness of the flakes was estimated as ~3.6 nm by atomic force microscopy, which indicated the presence of five layers (Supporting Information Figure S1). Large patterns formed through photo-lithography for each device, and fine electrodes for source and drain were completed after electron beam-lithography. The source–drain contacts of Cr/Au (10/80 nm) were deposited via thermal evaporation. Electrical transport measurements were performed using a Keithley 2400 source meter (Beaverton, OR, USA) and a Keithley 6485 picoammeter (Beaverton, OR, USA). The final optical device image is illustrated in Figure 1(b). All of the measurements were carried out at room temperature. Raman spectra were recorded with a Renishaw microspectrometer equipped with a 514 nm laser at room temperature. DUV light (*λ* = 220 nm and average intensity of 11 mW cm^−2^) was used for illumination.

A 10-nm-thick Al_2_O_3_ passivation layer was deposited on the FL-MoS_2_ at 200 °C through atomic layer deposition (ALD; Lucida D100 ALD by NCD Co., Ltd, Daejeon, Korea). During the growth process, 100 cycles with a growth rate of ~1 Å per cycle were completed to reach the desired thickness. Trimethylaluminum and deionized water were used as precursors. Ultra-pure N_2_ (99.999%) was used as a carrier and purging gas. The pressure of the growth chamber was maintained at ~5.5 × 10^−3^ torr during deposition.

## 3. Results and discussion

Raman spectroscopy is a fast and reliable tool that provides information about structural and local perturbation of 2D materials. The Raman spectrum of MoS_2_ is dominated by two vibrational modes: E^1^_2 g_, which is attributed to in-plane vibrations of two S atoms with respect to the Mo atom, and A^1^ _g_, which corresponds to the out-of-plane vibrations of S atoms in opposite directions. The E^1^_2 g_ and A^1^ _g_ modes are sensitive to the number of layers forming the structure of MoS_2_.[17, 18] The Raman spectra of FL-MoS_2_ layers were recorded at room temperature with a laser excitation of ~514 nm and a small laser power of ~1.0 mW to avoid the heating effect. Figure 1(c) shows the Raman spectra of pristine and gas-treated (in the dark and under DUV light) FL-MoS_2_ (five layers; see Figure S1). The FL-MoS_2_ exhibited strong bands at ~383.2 and ~407.7 cm^−1^, which is ascribed to in-plane vibrational (E^1^_2 g_) and out-of-plane vibrational (A_1 g_) modes, respectively. The vibrational modes of FL-MoS_2_ did not significantly change after treating the samples with O_2_ and N_2_ gases in the dark. However, we observed a shift toward a lower wave number after DUV treatment of FL-MoS_2_ in the presence of N_2_ gas. Subsequently we observed a shift toward a higher wave number in the presence of O_2_ gas under DUV light (Figure 1(c)). After the DUV treatment in N_2_ gas, the red shift of A_1 g_ and E^1^_2 g_ was ~2.2 and ~1.6 cm^−1^, respectively. The subsequent DUV treatment in O_2_ gas environment made the blue shift of A_1 g_ and E^1^_2 g_ by ~2.1 and ~1.5 cm^−1^, respectively. The red and blue shifts in peak (E^1^_2 g_ and A_1 g_) position are due to doping of electrons and holes on FL-MoS_2_ by DUV treatment in N_2_ and O_2_ gas, respectively. The mechanism of Raman shifts by adsorption and desorption of these gas molecules is attributed to electron-phonon coupling of modes. The A_1 g_ mode couples more strongly with electrons than the E^1^_2 *g*_ mode. The electron doping in MoS_2_ causes softening specifically of its Raman-active A_1 g_ phonon, accompanied by increase in line-width of its Raman peaks.[19] In contrast, the other Raman mode with E^1^_2 g_ symmetry is less sensitive to electron doping. The Raman spectrum for each case was obtained from five different points on the MoS_2_ surface, and the results were consistent.

**Figure 2.**
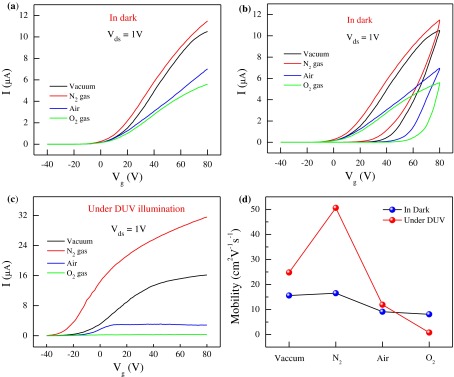
Electrical transport of FL-MoS_2_ FET before passivation. (a) Transfer characteristics of the FL-MoS_2_ FET device in the dark. Transfer characteristics were in vacuum, N_2_, air, and O_2_ environments. (b) Hysteresis in transfer characteristics of the FL-MoS_2_ FET device in the dark. Transfer characteristics were obtained under various environmental conditions (vacuum, N_2_ gas, air, and O_2_ gas). (c) Transfer characteristics of the FL-MoS_2_ FET under DUV illumination in vacuum, N_2_, air, and O_2_ environments. The power intensity and wavelength of light were 11 mW cm^-2^ and 220 nm, respectively. (d) Field-effect mobility of the FL-MoS_2_ FET device in the dark and in the presence of DUV illumination under various environmental conditions.

The electrical transport measurements of the FL-MoS_2_ FETs (sample-1; L = 8.1 μm and W = 7.6 μm) were carried out under various environmental conditions. Transfer characteristics (drain–current I_D_ as a function of back-gate voltage *V*_*g*_) were measured at room temperature with fixed source–drain voltage (*V*_*ds*_) = 1 V. Figure 2(a) presents the transfer characteristics of the FL-MoS_2_ FETs in the dark under various environmental conditions. The measurements were performed in vacuum, followed by N_2_, air, and O_2_ gas environment. The current level of the device was almost similar in the N_2_ and vacuum environments but was substantially reduced in the air and O_2_ environments. The I_on_/I_off_ of our device was ~10^5^ and remained almost same in vacuum, N_2_, air, and O_2_ gas flow (Supporting Information Figure S2a).

Figure 2(b) illustrates the hysteresis in the transfer characteristics of FL-MoS_2_ in the dark under different environmental conditions. We swept the *V*_*g*_ from –40 to 80 V repeatedly and observed a hysteresis in vacuum. Later on, the same devices were exposed in N_2_ gas and found a similar hysteresis trend to that in vacuum. However, further exposure of devices to air and oxygen significantly changed the hysteresis because of adsorption of oxygen atoms/molecules on top of MoS_2_ surface.[20] This kind of oxygen adsorption resulted in formation of charge-trapping sites and made the hysteresis prominent in FL-MoS_2_ FETs. In transfer characteristics in Figure S2a, we found that the threshold voltages (V_th_) of FL-MoS_2_ FETs was near –35, –37, –34, and –28 V in vacuum, N_2_, air, and O_2_, respectively. Furthermore, we observed that the V_th_ remained similar in vacuum and N_2_ gas but shifted toward positive gate voltages when the devices were exposed to air and O_2_ gas. The shift of V_th_ to positive back gate voltages indicates an electron deficiency in FL-MoS_2_ due to exposure to air and oxygen. Such behavior was previously reported for MoS_2_-related FET devices in O_2_ environments, which was also attributed to the absorption of oxygen molecules into sulfur or defect states on the MoS_2_ surface, which traps the charge carriers.[21, 22]

**Figure 3.**
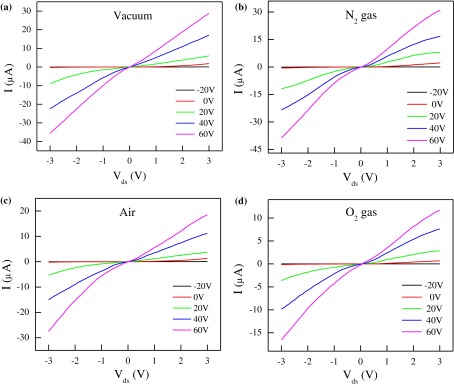
Drain-source characteristics of the device in the dark. The output characteristics (*I*_D_-*V*_ds_) of the FL-MoS_2_ FET at fixed *V*_*g*_ in (a) vacuum, (b) N_2_, (c) air, and (d) O_2_ environments. *V*_*g*_ ranges from –20 V to + 60 V with a step of 20 V.

Figure 2(c) shows the transfer characteristics of the FL-MoS_2_ FETs under various environmental conditions in the presence of DUV light. DUV light substantially improved the drain current of the FL-MoS_2_ FETs in N_2_ and in vacuum, but significantly decreased it in air and in O_2_ gas environments. DUV light reduced the oxygen atom/molecules from the MoS_2_ nanosheet surface in N_2_ and vacuum environments. The adsorption of oxygen atom/molecules on the MoS_2_ nanosheet reduced the number of charge carriers (electrons), causing a decline of drain current, which is consistent with previous reports.[23, 24] Figure 2(d) shows the field-effect mobility (*μ*) of the FL-MoS_2_ device under various environmental conditions in the dark and in the presence of DUV light. The mobility (*μ*) of the FL-MoS_2_ device was obtained using the following relation:

(1) $$\mu =\frac{L}{WC_{g}V_{\text{d}s}}\frac{\text{d}I_{ds}}{\text{d}V_{g}}$$

where d*I*_*ds*_/d*V*_*g*_ is slope of the transfer curve in the linear region, *C*_*g*_ is the gate capacitance (~115 aF μm^−2^) of Si/SiO_2_ substrates (300 nm), *V*_*ds*_ is the source–drain current for each device, *L* is the channel length, and *W* is the channel width. In the dark, we estimated the *μ*_vacuum_, *μ*_N2_, *μ*_air_, and *μ*_O2_ of the FL-MoS_2_ FETs to be ~15.6, 16.5, 9.1, and 8.1 cm^2^ V^−1^s^−1^, respectively. However, under DUV illumination, the *μ*_vacuum_, *μ*_N2_, *μ*_air_, and *μ*_O2_ of the FL-MoS_2_ FETs were ~24.8, 50.6, 11.9, and 0.8 cm^2^ V^−1^s^−1^, respectively. The FL-MoS_2_ FETs exhibited a high I_D_ and large mobility in N_2_ but showed a small I_D_ in air and O_2_ environment under DUV illumination.[25] Figure 3 presents the output characteristics of the FL-MoS_2_ FETs in vacuum, N_2_, air, and O_2_ environments in the dark. The output characteristics (I_D_ versus *V*_ds_) were measured at a fixed *V*_*g*_ ranging from −20 V to + 60 V with a step of 20 V. The nonlinear I-V characteristics supports that the Cr/Au metal electrodes make Schottky barrier due to the work function difference between metal and MoS_2_.

**Figure 4.**
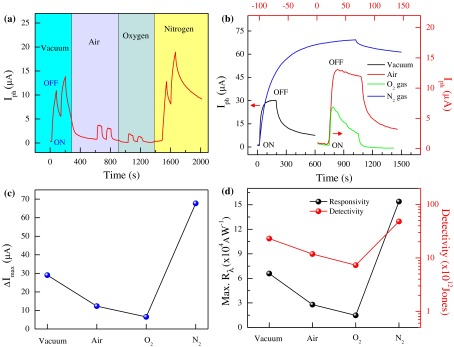
Photocurrent response in various environments. (a) Time-dependent photocurrent response of the device in different environments at *V*_*ds*_ = 1 V and *V*_*g*_ = 0 V. DUV light was first turned on for 60 s and then turned off for 60 s. (b) Photocurrent saturation of the FL-MoS_2_ FET in vacuum and N_2_ gas (left axis and bottom axis), and in air and O_2_ gas (right axis and top axis) at (*V*_*ds*_ = 1 V and *V*_*g*_ = 0 V). (c) Saturated photocurrent values in vacuum, N_2_ gas, air, and O_2_ gas. (d) Modulation of photocurrent response parameters; maximum responsivity (left axis) and detectivity (right axis) in different environments.

To measure the photodetector response, the MoS_2_ nanosheet device was irradiated with DUV light (sample-2; L = 1.3 μm and W = 3.15 μm). In Figure 4(a) we show the transient current response to a cycle of 60 s with alternating ON and OFF of the light source (*V*_*ds*_ = 1 V and *V*_*g*_ = 0 V) under various environmental conditions. Figure 4(a) illustrates that the photocurrent response depended on various environments. The photocurrent response was weak in O_2_ gas but strong in N_2_ gas. The MoS_2_ nanosheet exhibited a repeatable and reasonably stable response to incident DUV light under various environmental conditions. We also found that the photocurrent response increased with *V*_*ds*_. The photocurrent (Δ*I*_*ph*_ = *I*_*ph*_ – *I*_dark_) after 60 s DUV illumination reached ~14.7, 39.7, 83.3, and 88.5 μA at *V*_*ds*_ = 1, 4, 6, 8 V, respectively (Supporting Information Figure S2b and c).

In Figure 4(b), the photocurrent saturation was examined under various environmental conditions. We illuminated the FL-MoS_2_ FETs with DUV light until the photocurrent saturation was obtained and then turned off the DUV light to observe photocurrent decay behavior. The maximum photocurrent values (Δ*I*_max_ = *I*_saturation_ – *I*_dark_) of the FL-MoS_2_ FETs were ~29, 12.3, 6.5, and 67.7 μA (*V*_*ds*_ = 1 V and *V*_*g*_ = 0 V) under various environmental conditions (Figure 4(c)). The largest and smallest photocurrent saturations were observed in N_2_ and O_2_ gas environments, respectively.

Two key figures of merit were measured to quantify detector performance. Current responsivity (*R*_*λ*_) and detectivity (*D* ^*^) were defined as follows [26]:

(2) $$R_{\lambda }=\frac{I_{ph}}{PA}$$

(3) $$D^{*}=\frac{R_{\lambda }A^{\frac{1}{2}}}{\sqrt{\left(2eI_{\text{dark}}\right)}}$$

where Δ*I*_*ph*_ is the photo-excited current, *P* is the light power intensity, and *A* is the effective area of the photodetector. Responsivity is defined as the photocurrent generated per unit power intensity of the incident light on the effective area of a photoconductor. The power intensity of our DUV incident light and the area of device were 11 mW cm^−2^ and 4.09 μm^2^, respectively. In Equation (3), *e* is the absolute value of the electron charge and *I*_dark_ is the current density in dark. Figure 4(d) shows the responsivity and detectivity of our devices, where *R*_*λ*_ was estimated to be ~65.9, 27.9, 14.7, and 154 kAW^−1^ in vacuum, air, O_2_, and N_2_ gas environments, respectively. *D* ^*^, which is measured in Jones units (1 Jones = 1 cm Hz^1/2^ W^−1^), ranged from ~10^13^–10^14^. These data indicated the best response in N_2_ gas, which is more than ~10^4^ times higher than that of the previously reported 2D, layered material devices on Si/SiO_2_ substrates.[26–30]

**Figure 5.**
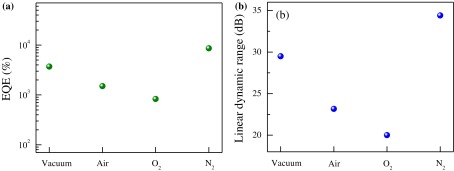
Light detection response. Crucial factors to be calculated in vacuum, N_2_, air, and O_2_ for (a) external quantum efficiency and (b) linear dynamic range. The data are obtained from Figure 4(b), (c), and (d).

To investigate photodetector performance further, two other critical parameters, namely EQE and LDR (typically presented in the unit of dB), were measured as seen in Figure 5(a) and (b), respectively. The EQE (= hc*R*_*λ*_/e*λ*, where h is a plank constant, c is the speed of light, *R*_*λ*_ is the responsivity at a DUV wavelength of 220 nm, and e is the electron charge) is defined as the number of electron–hole pairs excited by one absorbed photon. The EQE was estimated to be ~3700, 1500, 830, and 8600% in vacuum, air, O_2_, and N_2_ environments, respectively. Compared with previously reported devices, our MoS_2_ devices on the Si/SiO_2_ substrate exhibited higher EQE in N_2_ gas.[24,28,31,32] There are three factors which improve external quantum efficiency (EQE) of our devices; higher responsivity, light of lower wavelength and N_2_ gas environment. The wavelength, *λ*, is an important factor which is the denominator in the formula (EQE = hc*R*_*λ*_/e*λ*). We enhanced our EQE to 8600% using smaller wavelength together with N_2_ gas environments. The N_2_ gas provides supportive environment for photocurrent generation. The LRD (= 20 log (*I*_*ph*_/*I*_dark_), where *I*_*ph*_ is the photocurrent measured at a light intensity of 11 mW cm^−2^) was measured under DUV illumination. The measured LDR values of the MoS_2_ nanosheet device were ~29.5, 23.16, 20, and 34.4 dB in vacuum, air, O_2_, and N_2_ environments, respectively. The results demonstrate that MoS_2_ nanosheet devices in N_2_ gas can be potential photodetectors in the future.

**Figure 6.**
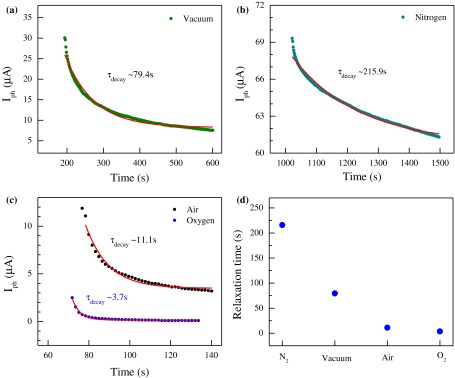
Photocurrent decaying behavior in different environments. Transient property of FL-MoS_2_ FETs under different conditions and data fitting (red line) with a single exponential equation. (a) Decay of drain current in vacuum. (b) Decay of drain current in N_2_. The device exhibits a slow decay with a large time constant. (c) Decay of drain current in air and O_2_. The drain current decays with a small time constant. (d) Relaxation time constants of the device in vacuum, N_2_, air, and O_2_.

Relaxation response time or decaying behavior is another key factor in photodetector performance. Decaying behavior is observed after photocurrent saturation under various environmental conditions. Relaxation data were extracted from Figure 4(b), when light was turned off after photocurrent saturation. The response time obtained from some 1D nanostructures or graphene oxide-based photodetectors ranges from seconds to several tens of minutes.[33–35] The wide range of response time is attributed to the difference in the materials or device structures. The dynamic response to DUV illumination for relaxation time can be expressed as [28, 36]:

(4) $$I_{\text{ph}}\left(t\right)=I_{\text{dark}}+A\text{exp}\left(-\frac{t}{\tau_{\text{decay}}}\right)$$

where *A* is a scaling constant, *τ*_decay_ is a time constant for decaying, and *t* is the time after DUV light is switched on or off. The time constant (*τ*) can be calculated by fitting the experimental data. Figure 6(a–c) describes the decaying photocurrent behavior in N_2_, vacuum, air, and O_2_ environments, respectively, and the red lines indicate the fitting data. The relaxation time was calculated to be ~215.9, 79.4, 11.1, and 3.7 s in N_2_, vacuum, air, and O_2_, respectively (Figure 6(d)). These results demonstrated that the decay process was very slow in N_2_ and significantly fast in O_2_ gas. Thus, the adsorbates and defect states originating from ambient air, water, and oxygen atoms/molecules play an important role in photocurrent relaxation and electron-hole pair recombination dynamics. In general, the surface-adsorbed oxygen significantly affects the photoresponse of MoS_2_ and ZnO films.[37,38] In the present study, electron-capturing impurity states were reduced under DUV light in N_2_ gas environment but were enhanced under DUV light in O_2_ gas environment. Thus, the decay time was significantly longer in N_2_ environment and substantially shorter in O_2_ environment. Evidently, decaying behavior was robustly dependent on the surrounding environment.

**Figure 7.**
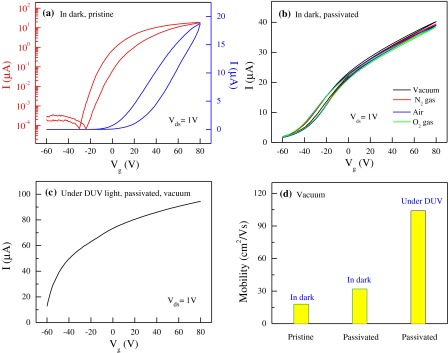
Electrical transport measurements of passivated FL-MoS_2_ FET. (a) Transfer characteristics of the FL-MoS_2_ FET device in the dark. Transfer characteristics are measured in vacuum. The linear scale is on the right axis, and the logarithm scale is on the left axis. (b) Hysteresis in transfer characteristics after passivation. The measurements were performed in the dark under different environmental conditions with *V*_*g*_ from –60 V to +80 V. (c) Transfer characteristics under DUV illumination. The measurements were carried out in vacuum at *V*_*ds*_ = 1 V. The power intensity and wavelength of light were 11 mW cm^–2^ and 220 nm, respectively. (d) Field–effect mobility of the passivated FL-MoS_2_ FET device in the dark and under illumination. The measurement was conducted in vacuum.

We measured the electrical transport of the FL-MoS_2_ FETs (sample-3; L = 1.5 μm and W = 1.2 μm) in vacuum at a bias voltage *V*_*ds*_ = 1 V to study the passivation effect by high-k dielectric materials to circumvent the environmental effects. The transfer characteristic of the pristine device at room temperature in the dark is shown in Figure 7(a), where a pronounced hysteresis can be observed. A 10-nm-thick passivation layer of Al_2_O_3_ was deposited on the same device through atomic layer deposition (ALD). After depositing the passivation layer, the transfer characteristics of the device were examined in the dark under various environmental conditions (Figure 7(b)). The MoS_2_ device was almost hysteresis-free with increased drain current and exhibited n-type. The Al_2_O_3_ layer on the MoS_2_ surface served as a protecting or capping layer. Hence, the effect of external environments was effectively disregarded.

In addition, field-effect mobility increased after passivation by Al_2_O_3_ film. The improvement of device performance after deposition of a high-k dielectric is associated with the suppression of Coulomb scattering, modification of phonon dispersion, the difference of Al_2_O_3_ and SiO_2_ dielectric constants (3.9 for SiO_2_ and 9.0 for Al_2_O_3_), and the removal of impurities during ALD growth at 200 °C.[39–41] Extensive theoretical work including the calculation of phonon dispersion relations in MoS_2_, the calculation of scattering rates on phonons and charge impurities, is necessary to describe these phenomena completely. After the deposition of a 10-nm-thick Al_2_O_3_ layer, the electrical properties were also measured under DUV illumination in vacuum at *V*_*ds*_ = 1 V. A huge increment in drain current was observed (Figure 7(c)). The field-effect mobility of the pristine and passivated devices in the dark and in the presence of DUV illumination was measured in vacuum, and the mobility was ~18, 32, and 104 cm^2^ V^−1^s^−1^, respectively (Figure 7(d)).

**Figure 8.**
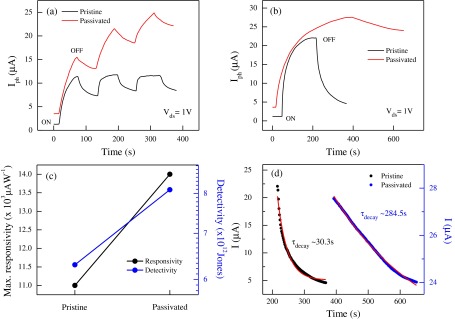
Photocurrent response. (a) Time-dependent photocurrent response of the pristine and passivated devices under vacuum at *V*_*ds*_ = 1 V and *V*_*g*_ = 0 V. DUV light was initially turned on for 60 s and then turned off for 60 s. (b) Photocurrent saturation of FL-MoS_2_ FETs in vacuum for the pristine and passivated devices at *V*_*ds*_ = 1 V and *V*_*g*_ = 0 V. (c) Photocurrent response parameters, including maximum responsivity (left axis) and detectivity (right axis), of the pristine and passivated devices. The data were obtained from Figure 8(b). (d) Decaying behavior of the pristine and passivated devices when the light was turned off after photocurrent saturation. The red line presents the fitting by a single exponent equation.

We explored the photoresponse of the FL-MoS_2_ FETs (sample-4; L = 2 μm and W = 8.2 μm) passivated by the Al_2_O_3_ layer (10 nm). The transfer characteristics of the device are shown in Figure S2d (Supporting Information). The time-dependent response under DUV light is described in Figure 8(a) with switching light ON and OFF for a 60 s cycle at (*V*_*ds*_ = 1 V and *V*_*g*_ = 0 V) in vacuum. As shown in Figure 8(a), the passivated device exhibited a slow response because of the suppression of impurities and defect states by the Al_2_O_3_ layer. However, the photocurrent of the passivated device enhanced compared with that of the pristine device. In Figure 8(b), the maximum photocurrent saturation values (ΔI_max_ = I_saturation_ – *I*_dark_) of the pristine and passivated FL-MoS_2_ FETs were found to be ~20.6 and 24.2 μA, respectively. *R*_*λ*_ and *D* ^*^ were also investigated as shown in Figure 8(c). The maximum responsivity and detectivity of the pristine device were ~11 × 10^3^ AW^−1^ and ~6.3 × 10^12^ Jones, respectively. However, those after deposition of the Al_2_O_3_ layer were ~14 × 10^3^ AW^−1^ and ~8.2 × 10^12^ Jones, respectively. To investigate further the effect of passivation, the relaxation time (*τ*_decay_) of the pristine and passivated devices was examined (Figure 8(d)). The data for decaying time were obtained from Figure 8(b) and exponentially fitted by the single exponential Equation (4). The carrier lifetimes for the pristine and passivated devices were ~30.3 and 284.5 s, respectively. As depicted in Figure 8(d), the relaxation time of the passivated device was significantly longer than that of the pristine device. This result can be attributed to the fact that the intermediate states of defects and oxygen constitutes are reduced after Al_2_O_3_ deposition and those excited electrons take a long time to relax.

## 4. Conclusions

In conclusion, we fabricated FL-MoS_2_ FETs and studied the effects of environmental gases in the dark and under DUV illumination. We determined that DUV light induced red and blue shifts of Raman peak (E^1^_2 g_ and A_1 g_) positions in the presence of N_2_ and O_2_ gases, respectively. We investigated the electrical transport measurements of FL-MoS_2_ FETs under various environmental conditions and found that device performance did not degrade in N_2_ gas in the dark. However, O_2_ gas significantly reduced the ON-current state. In the dark, the environmental gases did not modify the I_on_/I_off_ of the device and remained consistent at ~10^5^. The threshold voltage and hysteresis were nearly similar in vacuum and N_2_ gas but shifted toward positive gate voltage accompanied with a large hysteresis loop in air and O_2_ gas. However, the environmental gases changed the characteristics of the FL-MoS_2_ FETs under DUV illumination. The photocurrent response and saturated photocurrent of the FL-MoS_2_ FETs under various environmental conditions were examined to investigate light detection parameters, such as responsivity, detectivity, external quantum efficiency, and linear dynamic range. The results demonstrated a strong photoresponse in N_2_ gas but a poor photoresponse in O_2_ gas. The photocurrent relaxation after turning off DUV light was also examined under various environmental conditions. The lifetime of a carrier was longer in N_2_ gas but shorter in O_2_ gas, indicating that N_2_ gas helped the device recover from defects and impurities, while O_2_ gas adversely affected device characteristics. Furthermore, we passivated the device by depositing the Al_2_O_3_ layer to protect MoS_2_ from environmental effects. We observed almost hysteresis-free transfer characteristics with improved electrical and photoelectrical responses and field-effect mobility after deposition of the passivation layer. We also found that *τ*_decay_ increased after Al_2_O_3_ layer deposition. This phenomenon may be attributed to the screening of impurities and defects from the MoS_2_ surface. Thus, we developed improved, stable, reliable, and hysteresis-free FL-MoS_2_ FETs for various nanotechnology applications.

## Disclosure statement

No potential conflict of interest was reported by the authors.
